# Regarding the Utility of Unstructured Data and Natural Language Processing for Identification of Breast Cancer Recurrence

**DOI:** 10.1200/CCI.21.00091

**Published:** 2021-10-12

**Authors:** Debra P. Ritzwoller, Michael J. Hassett, Hajime Uno

**Affiliations:** Debra P. Ritzwoller, PhD, Institute for Health Research, Kaiser Permanente Colorado, Aurora, CO; Michael J. Hassett, MD, MPH, Department of Medical Oncology, Dana-Farber Cancer Institute, Boston, MA, Harvard Medical School, Boston, MA; and Hajime Uno, PhD, Harvard Medical School, Boston, MA

## TO THE EDITOR:

In the companion to this article, Karimi et al^1^ described the use of natural language processing (NLP) to identify distant cancer recurrence, along with the timing and the specific site of distant recurrence, for patients with breast and hepatocellular carcinomas. We commend the authors for developing techniques that identify the specific site of recurrence using unstructured data derived from the electronic medical record at one health care system. However, we believe that the manuscript does not address important methodologic limitations and does not provide sufficient details regarding key results. Moreover, to fully grasp the potential impact of this study, it is important to place its findings in a broader context that considers previously developed recurrence detection methods and the practical constraints of electronic health record (EHR) data.

First, the authors describe their technique as being NLP-specific, contrasting their approach to algorithms that rely on structured standardized codes (eg, International Classification of Diseases [ICD], pharmacy, etc). However, the cohorts used in this study were subsets of patients with cancer specifically selected on the basis of their high likelihood of having recurrence. Moreover, a priori identification of these high-risk patients relied on the use of structured data codes (eg, inclusion into the breast cancer cohorts was dependent on specific procedure and pharmacy codes, such as having either undergone a prior surveillance mammogram followed by two computed tomography or magnetic resonance examinations or having received two systemic therapies). Thus, the approach is not NLP-only, but rather a hybrid technique that combines NLP with claims- or EHR-based codes. The metrics reported in the manuscript only assess the performance of the NLP component within the enriched high-risk cohort; the results may be biased or not be representative of the algorithm's performance in a larger or unselected cancer cohort. We suggest that the correct denominator for assessing algorithm performance should be the full sample of 7,116 patients, and that the algorithm should include both the codes used to select high-risk patients and the NLP model to identify recurrence.

Second, the investigators included stage IV breast cancer cases in their analysis. These patients have de novo distant metastatic disease and, in nearly all situations, will never become disease-free. So, they cannot be eligible to recur. If the algorithm were to identify events in these patients, these events would be better classified as disease progression rather than cancer recurrence.

Third, the investigators compared performance of their hybrid model with that of a claims-based approach that relied only on a small group of ICD codes. Previous studies have demonstrated that this is an inferior approach that does not reflect current practice.^2^ Published studies have also described methods that use standardized codes to detect cancer recurrence and the timing of recurrence across multiple cancer sites, including breast, colorectal, and lung cancer.^3-5^ Validation of these recurrence detection or timing algorithms, which have used multiple data sets and large patient populations, has demonstrated that these algorithms offer a recurrence detection sensitivity of ≥70% and an area under the receiver operating characteristic curve of ≥ 0.900. Moreover, these published algorithms have proven to be valuable for addressing a variety of novel and timely cancer outcome research questions using public and open data sources.^6-10^

Fourth, this study found that 2.31% of recurrent patients had target ICD codes. This value is extremely low and seems to be inconsistent with previous research studies, suggesting either a major omission of key codes or that the data set used for this analysis is not representative of data sets derived from other health care system EHRs or Medicare claims.^8^

Fifth, the authors underscore the superiority of their findings regarding estimating recurrence timing, yet the manuscript provides little description of the recurrence timing model methods or results. If the authors are referring to specific results from their previous manuscript, additional clarification would be illustrative.

Finally, it seems likely that NLP techniques using unstructured text data will be superior to recurrence detection or timing algorithms that rely on structured EHR or claims data. However, NLP-based techniques have important shortcomings when used in the real-word setting: (1) access to unstructured text data and computing resources may be limited and (2) NLP-based solutions may be less portable between data sources because of variability in the way that clinicians document unstructured text. For all these reasons, we believe that EHR- or claims-based recurrence detection algorithms will continue to be useful for researchers for the foreseeable future.
